# The Lnc‐ENST00000602558/IGF1 axis as a predictor of response to treatment with tripterygium glycosides in rheumatoid arthritis patients

**DOI:** 10.1002/iid3.1098

**Published:** 2024-01-16

**Authors:** Yang Gao, Xiaoyue Wang, Yanfeng Gao, Jian Bai, Yanpeng Zhao, Renyi Wang, Hanzhou Wang, Guangzhao Zhu, Xixi Wang, Xiaochen Han, Yanqiong Zhang, Hailong Wang

**Affiliations:** ^1^ Department of Chinese Medicine Tsinghua University Hospital Beijing China; ^2^ Institute of Chinese Materia Medica China Academy of Chinese Medical Sciences Beijing China; ^3^ Department of Dermatology The Second Mongolian Medical Hospital of Traditional Chinese Medicine Chi Feng City Inner Mongolia China; ^4^ Guizhou University of Traditional Chinese Medicine Graduate School Guiyang City Guizhou China; ^5^ Department of Rheumatology, Guang'anmen Hospital China Medical Sciences Beijing China; ^6^ Department of Rheumatology Qinghai Hospital of TCM Xining City Qinghai China; ^7^ Department of Internal Medicine Beijing Fengsheng Hospital of Traditional Medical Traumatology & Orthopedics Beijing China; ^8^ Department of Rheumatology, Dongzhimen Hospital Beijing University of Chinese Medicine Beijing China

**Keywords:** IGF1, Lnc‐ENST00000602558, partial‐least‐squares, rheumatoid arthritis, tripterygium glycoside

## Abstract

**Aims:**

Growing clinical evidence suggests that not all patients with rheumatoid arthritis (RA) benefit to the same extent by treatment with tripterygium glycoside (TG), which highlights the need to identify RA‐related genes that can be used to predict drug responses. In addition, single genes as markers of RA are not sufficiently accurate for use as predictors. Therefore, there is a need to identify paired expression genes that can serve as biomarkers for predicting the therapeutic effects of TG tablets in RA.

**Methods:**

A total of 17 pairs of co‐expressed genes were identified as candidates for predicting an RA patient's response to TG therapy, and genes involved in the Lnc‐ENST00000602558/GF1 axis were selected for that purpose. A partial‐least‐squares (PLS)‐based model was constructed based on the expression levels of Lnc‐ENST00000602558/IGF1 in peripheral blood. The model showed high efficiency for predicting an RA patient's response to TG tablets.

**Results:**

Our data confirmed that genes co‐expressed in the Lnc‐ENST00000602558/IGF1 axis mediate the efficacy of TG in RA treatment, reduce tumor necrosis factor‐α induced IGF1 expression, and decrease the inflammatory response of MH7a cells.

**Conclusion:**

We found that genes expressed in the Lnc‐ENST00000602558/IGF1 axis may be useful for identifying RA patients who will not respond to TG treatment. Our findings provide a rationale for the individualized treatment of RA in clinical settings.

## 1 INTRODUCTION

*Tripterygium wilfordii* is a well‐known Chinese traditional herbal medicine.^1^ The roots of *T. wilfordii* contain several natural active components, including terpenes, alkaloids, organic acids, polysaccharides, phenols, lignins, and amino acids.^2^ Three of the active components from *T. wilfordii*, included triptolide, celastrol, and multiglycoside, have attracted considerable attention.^3^ Tripterygium glycoside (TG) is one of the primary biologically active ingredients obtained from the root of *T. wilfordii*. The refinement process consists of peeling, crushing, ethanol extraction, chloroform extraction and separation by silica gel column chromatography. The major chemical components of TG tablet are sesquiterpenes, diterpenes, triterpenes, alkaloids, flavonoids, glycosides, organic acids, and other constituents.^4^

TG is a traditional Chinese medicine used to treat rheumatoid arthritis (RA).^5^, ^6^ TG is also considered to be a potential chemical scaffold for developing new drugs for RA treatment because of its significant effect in slowing RA progression.^7^, ^8^, ^9^ Clinical investigations have shown that the therapeutic effect is TG is better than that of several other anti‐rheumatic drugs.^5^, ^10^, ^11^, ^12^ However, >30% of RA patients who receive TG fail to show significant clinical improvement. This lack of efficacy may be due to a combination of epigenetic, physiological, environmental, and especially genetic factors that lead to significant individual differences in treatment response.^3^, ^8^, ^13^, ^14^ The heterogeneity of RA patients in terms of pathological presentation, disease progression, and treatment response suggests that there are multiple disease‐related differences at the molecular level.^15^, ^16^, ^17^, ^18^ Researchers are currently attempting to identify various factors that affect an RA patient's treatment response, including various long noncoding RNAs (lncRNAs), genes, and proteins, to further promote individualized treatment and ensure its efficacy.^16^, ^19^, ^20^

In recent years, high‐volume genomic and transcriptome technologies, such as gene microarray technology for simultaneous and comprehensive detection of numerous gene expression profiles, have played an important role in identifying lncRNA and RA‐related gene interactions, as well predicting disease behavior and a patient's response to treatment.^7^, ^21^, ^22^ Although gene expression microarray assays have the advantages of high throughput and high sensitivity, those assays do not fully elucidate the entire biological process that regulates RA. In addition, differences in sample size and quality can cause inconsistencies among the numerous differential transcripts identified by microarrays.^8^, ^10^ To address these issues, we performed a molecular network analysis that integrated the high‐throughput benefits of gene expression profiling with our knowledge of drug–disease interactions. Next, the differential expression of paired genes was assessed, and important regulators were identified according to the differential expression patterns and network topological features. Subsequently, a partial least squares (PLS) model based on the expression of paired genes was constructed to predict the therapeutic effect of TG treatment. Lastly, the function of filtered paired‐expressed genes, which mediate how TG functions in RA treatment was further confirmed in rheumatoid arthritis synovial fibroblast (MH7a) cells.

## 2 MATERIALS AND METHODS

### 2.1 Patients

From October 2018 to October 2019, a total of 52 RA patients were recruited at a hospital to participate Division of Rheumatology of Guang'anmen Hospital. The 52 RA patients were assigned to two main groups, which each had two subgroups: discovery group (*n* = 12, 6 responders and 6 nonresponders) and validation group (*n* = 40, 25 responders and 15 nonresponders). The Affymetrix EG 1.0 Array genome‐wide expression profile assay was used to detect lncRNAs and mRNAs that were differentially expressed in peripheral blood mononuclear cells (PBMCs) and were related to the efficacy of TG tablets in the former subgroups. The latter groups were used to validate the expression of candidates by quantitative real‐time polymerase chain reaction (RT‐qPCR) assays.

Inclusion criteria of the RA patients based on the 2020 ACR/European League Against Rheumatism (EULAR) Criteria or the American College of Rheumatology (ACR) 1987 criteria for RA.^23^ In brief, (1) a symptom duration of less than 1 year; (2) RA patients with a symptom last less than a year or treatment of TG tables less than 4 weeks were not used; and (3) availability of clinical and laboratory parameters at initiation of TG tablets and after 12 weeks, as well as availability of peripheral blood samples. The inclusion criteria used for RA patients were based on either the 2020 ACR/EULAR Criteria or the ACR 1987 criteria for RA. All the RA patients were between 18 and 70 years old, and RA patients with a symptom lasting > 1 year or whose symptoms had been treated with TG tablets for < 4 weeks were excluded from the study. The patients received TG tablets (20 mg, oral administration, three times per day) on a continuous basis for 12 weeks. Patients who responded to treatment were defined as those who achieved ACR 20, otherwise, they were classified as nonresponders.

### 2.2 PLS model

A PLS model was constructed based on gene expression in the peripheral blood of RA patients, and a fivefold cross‐validation was used to evaluate the model's performance. Sequentially maximizing the covariance between the linear combination of predictors and the response variable served as the objective standard for constructing components in the model. In the matrix, P represents candidate genes, and N represents cases. In addition, Y represents the N×1 vector of response values (the indicator of responders or non‐responders). The components were constructed by maximizing the objective criterion based on the sample covariance. Thus, the weight vector W was satisfied with the following objective criterion.

$$W={\text{argmaxcov}}^{2}\left(X_{w},Y\right).$$

Subsequently, the weight coefficients of genes were calculated using a training data set. The candidates in the PLS model are indicated as: P = {Pi}, i = 1, 2, 3.

The PLS model score (S) is defined as:

$$S=\Sigma L_{\text{Pi}}\left(3\right)\times W_{\text{Pi}},i=1,2,3.$$

L_Pi_ represents the candidates' expression pi in the RA patients.

Subsequently, the training data set was input into the PLS model, and the cutoff value for which the largest area under the receiver operating characteristic (ROC) curve (AUC) was used to calculate the threshold value (T) of the AUC score. The PLS classifier was used to predict a responder when the score was > T. For the fivefold cross‐validation, the candidates in the discovery cohort were classified into two groups: a training data set and testing data set, respectively. A fivefold cross‐validation was performed due to the small sample size of the discovery cohort. The mean accuracy, sensitivity, specificity, and AUC values determined by the ROC curves were calculated using the following formulas:

$$\begin{array}{l} \text{Sensitivity}=\text{TP}/(\text{TP}+\text{FN});\\ \text{Specificity}\;=\text{TN}/(\text{TN}+\text{FP});\\ \text{Accuracy}\;=(\Sigma \text{TP}+\text{TN})/N; \end{array}$$

TP, TN, FP, and FN refer to the numbers of true positive, true negative, false positive, and false negative result components in a test, while *N* refers to the total number of predictive samples.

### 2.3 Gene expression profiling

Samples of peripheral blood (PB) were collected from RA patients treated with TG tables (*n* = 12). Density gradient centrifugation was used to isolate the PBMCs, which were subsequently washed in sterile phosphate‐buffered saline (PBS). Next, the total RNA in PBMCs was extracted and eluted with 15 μL of RNase‐free water. The gene expression profiles (include mRNAs and lncRNAs) of the responders and non‐responders to TG tablets were respectively detected using an Affymetrix EG1.0 array system. A total of 57 reliable RA targets were collected from the database DRUGBANK. mRNAs that were differentially expressed between responders and non‐responders were identified by using the criteria: log_2_ fold‐change and a *p*‐value < .05, as determined by the unpaired student's *t*‐test. The Database Visualization and Integrated Discovery software package (DAVID; http://david.abcc.ncifcrf.gov/home.jsp, version 6.7) was used to analyze gene function via pathway enrichment. Pathway data was obtained from the Kyoto Encyclopedia of Genes (KEGG; http://www.genome.jp/kegg).

### 2.4 Gene signal transduction network analysis

Reliable gene‐gene interaction data were obtained from the public database STRING (Search Tool for Known and Predicted Protein Interactions; version 10.0, http://string-db.org), and the aggregate evidence score was higher than the median of all scores. In our network, nodes refer to genes that were differently expressed between the responder and nonresponder populations, and the edges refer to the interaction between nodes. To determine biomarkers of candidate genes for TG tablets, we assessed the topological importance of each node by calculating the following four topological characteristics: (1) Node degree: the sum of the advantages of connecting nodes to me with other genes, which measures the related genes in relation to other gene networks; (2) internode: the importance of a node relative to other nodes in the network; and (3) node intimacy: the time required for the measurement information to propagate from node I to all other nodes in turn. The greater the degree/intermediate degree/intimacy of a node, the more important that node is in the network.

### 2.5 Cell lines and cell culture

MH7A cells (catalogue ID: BFN60805933) were obtained from BLUEFBIO. The cells were cultured in Dulbecco's modified Eagle's medium (DMEM) (Hyclone, SH30022.01B) containing 10% fetal bovine serum (FBS) (Gibco, 10099‐141) and 1% penicillin‐streptomycin (Procell, PB180120) at 37°C in a humidified atmosphere with 5% CO_2_. A series of dissolved TG tablet solutions (1.25, 2.5, 5, 10, 20, 40, 80, 160, 320, 640, and 1280 μg/mL) (Huangshi Feiyun Pharmaceutical Co., Ltd.) were incubated with cultured MH7A cells at 37°C for 24 h to examine their cytotoxic effects. For the construction of RA cell model, tumor necrosis factor (TNF)‐α (PEPROTECH, 300‐01A) was incubated with cultured MH7A cells at a final concentration of 10 ng/mL for 24 h.

### 2.6 Enzyme‐linked immunosorbent assay (ELISA) assays

The levels of interleukin (IL)‐1β, IL‐6, and TNF‐α from the cultured supernatant were assessed by ELISA assays.^24^, ^25^, ^26^ In brief, MH7A cells were transfected with the indicated short hairpin RNA (shRNA) or small interfering RNA (siRNA) for 24 h, and subsequently treated with TNF‐α combined with different concentrations of TG for another 24 h. After treatment, the culture supernatants were collected. Then, 100 μL supernatants were added into the ELISA plates and incubated for 1 h at room temperature. After incubation, the plates were washed for three times by PBS. Then, the horseradish peroxidase‐conjointed second antibody was incubated with plates for 1 h at connected. Finally, the TMB was added to test the IL‐1β, IL‐6, and TNF‐α levels. The levels of IL‐1β, IL‐6, and TNF‐α in the culture supernatants were assessed by ELISA assays. ELISA kits for IL‐1β (201‐LB), IL‐6 (D6050), and TNF‐α (210‐TA) were purchased from R&D Systems.

### 2.7 Cells transfection

A pLKO.1‐puro plasmid (GenePharma) was used to construct shRNA for Lnc‐ENST00000602558 knockdown, and the corresponding empty vector served as a negative control. A si‐IGF1 and control si‐RNA were purchased from hanghai GenePharma Co., Ltd. The transient transfection of shRNA or siRNA (both from Shanghai GenePharma Co., Ltd.) was performed in six‐well (or 96‐well) plates using Lipofectamine® 3000 (Invitrogen; Thermo Fisher Scientific, Inc.) according to the manufacturer's protocols. Briefly, MH7A cells were seeded in a six‐well (or 96‐well) plates at a concentration of 2 × 10^5^ (2 × 10^3^) cells/well, and shRNA or siRNA transfection was performed when the cells grew to 40%–60% confluence. All molecules were transfected at a concentration of 50 nM. After transfection, the culture plate was placed in a constant temperature incubator at 37°C and 5% CO_2_ for 48 h.

### 2.8 Fluorescence in situ hybridization (FISH)

MH7A cells were seeded into 12‐well slides and cultured in a DMEM medium containing 10% FBS. When the cells reached 80% confluence, they were treated with TNF‐α or PBS (Ctrl) combined or not combined with TG for 1 h. Next, the treated cells were fixed with 4% paraformaldehyde, blocked with PBS containing 5% goat serum, and permeabilized with 0.2% Triton X‐100/PBS for 30 min. The cells were then hybridized with a cy3‐labled Lnc‐ENST00000602558 probe (GenePharma) overnight. The next morning, the cells were washed with 20×SSC eluant (Servicebio) and then incubated with IGF1 antibody (Abcam, ab106836, 1:500) overnight at 4°C. The next morning, the slides were re‐warmed for 1 h at room temperature, and then incubated with a secondary conjugated fluorescent dye (Abcam, ab150129, 1:1000) at 37° for 1 h. After counterstaining with DAPI (Abcam, ab228549, 1:5000), images were captured with a Nikon Eclipse TS100 microscope equipped with Micro‐Manager 1.4.22 acquisition software.

### 2.9 RNA pulldown assay

The cells were homogenized in 200 μL of cell lysis buffer (10 mM Tris‐HCl, pH = 8.0, 10 mM NaCl, 3 mM MgCl_2_, 0.5% NP‐40, and a protease inhibitor cocktail); after which, the cell lysates were immune‐precipitated by using an RNA 3′ End Desthiobiotinylation Kit (Pierce Biotechnology). Protein‐A agarose beads (Invitrogen, 101006) were used to isolate the immunoprecipitates, which were subsequently reverse‐transcribed and then digested with protease K overnight at 65°C. The immune‐precipitated protein and input protein were examined by western blot analysis.^27^

### 2.10 Statistical analysis

All statistical analyses were performed using IBM SPSS Statistics for Windows, Version 19.0 software (IBM Corp.). Quantitative data are expressed as a mean value ± standard error of mean of results obtained from three independent experiments. Each experiment was performed using at least six samples per group. The two‐tailed, unpaired student's *t*‐test was used for pair‐wise comparisons of genotypes or treatments. One‐way analysis of variance (ANOVA) was used for comparing three or more groups, as indicated in the figure legends and elsewhere. To assess statistical significance, data from three or more independent experiments were analyzed by using Tukey's post hoc test with a confidence interval of 95% after ANOVA.

## 3 RESULTS

### 3.1 Identification of differentially expressed LncRNAs and mRNAs that responded to TG treatment

**Figure 1:**
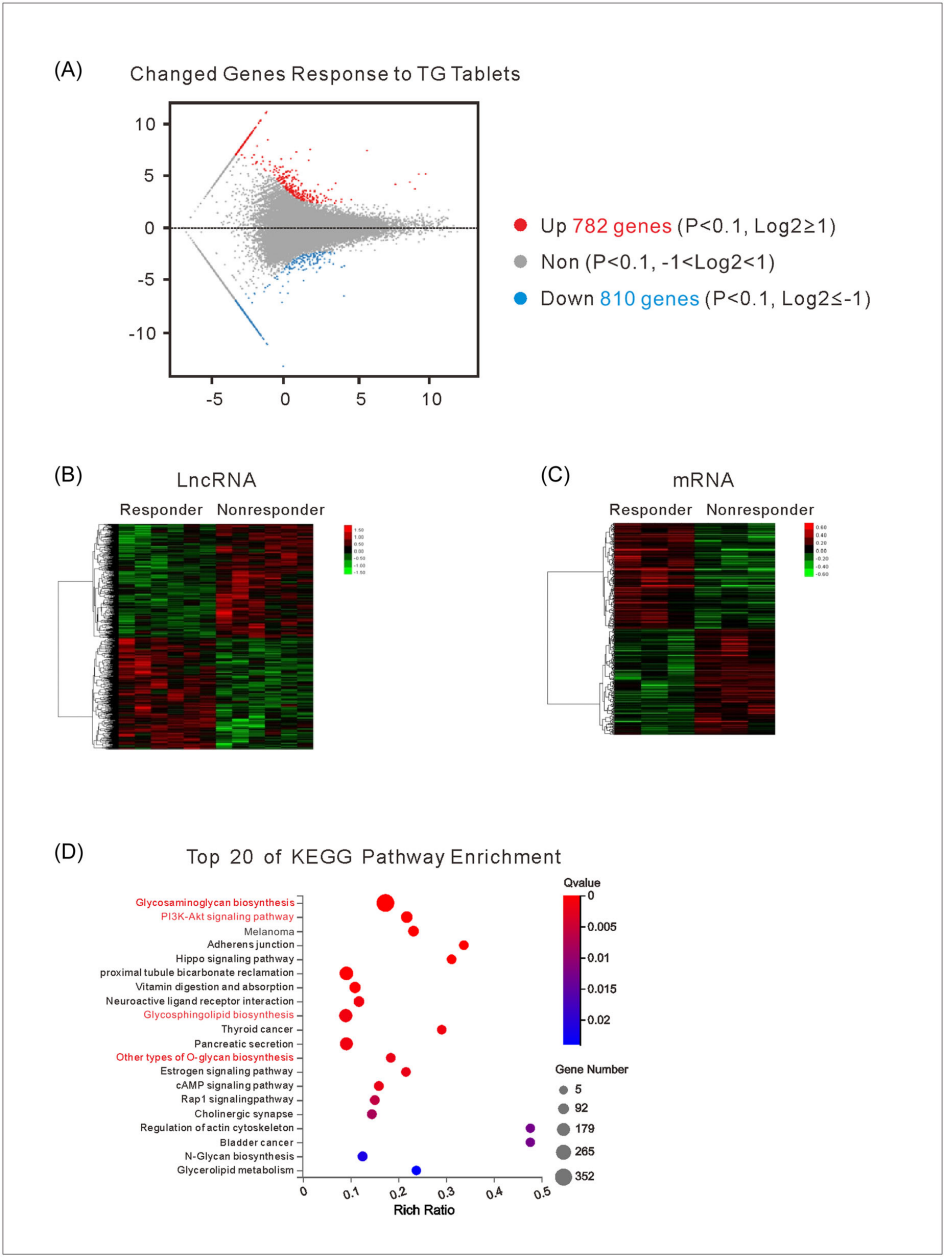
Identification of differentially expressed LncRNAs and mRNAs that responded to TG treatment. (A) Transcriptome changes in TG responder versus TG non‐nonresponder patients. Each dot represents a single gene transcript level as a fold‐change. Genes that were significantly upregulated are shown in red and genes that were significantly downregulated are shown in blue. (B and C) Heat map showing hierarchical clustering of lncRNAs (B) and mRNAs (C) that showed changes in the comparison between the responder (*n* = 6) and nonresponder (*n* = 6) patients. In the cluster analysis, red represents upregulated genes, and green represents downregulated genes. (D) KEGG pathway enrichment analysis of the commonly changed mRNAs in TG responder versus TG non‐nonresponder patients. The red column shows pathways related to inflammatory reactions. KEGG, Kyoto Encyclopedia of Genes and Genomes; lncRNAs, long noncoding RNAs.

To identify differentially expressed gene pairs related to the response to TG treatment of RA patients, we first compared the lncRNA expression profiles of RA patients who responded and did not respond to TG treatment (six patients per group). After examining a total of 1592 lncRNAs; among which 782 were upregulated and 810 downregulated, we found a significant difference in the differential expression of lncRNAs in the two groups (Figure 1A). Next, the profiles of the differentially expressed lncRNAs revealed distinctive patterns for responders and nonresponders to TG treatment as determined by unsupervised hierarchical clustering (Figure 1B). Similarly, as shown in heat maps (Figure 1C), we screened 212 mRNAs that showed significant expression characteristics related to the response to TG treatment of RA patients. Subsequently, a KEGG pathway enrichment analysis of the differentially expressed mRNAs was performed. Those results showed that the changed genes were significantly enriched in biological processes and pathways related to glycosaminoglycan biosynthesis and PI3K‐Akt signaling, suggesting that genes associated with a patient's response to TG treatment were also associated with the immune response (Figure 1D).

### 3.2 Identification of paired expressed genes based on the discovery cohort

**Figure 2:**
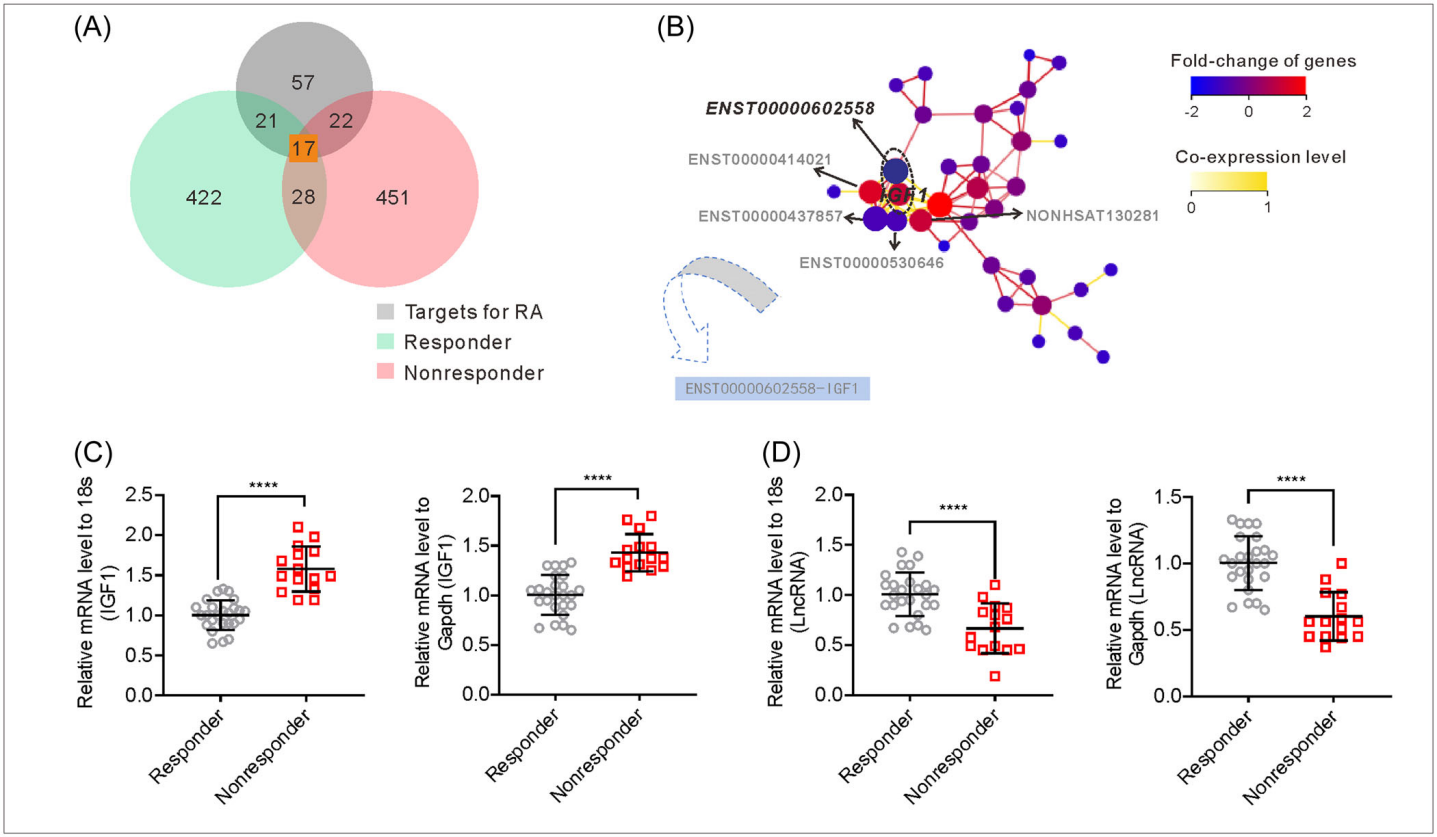
Identification of candidate biomarker genes that predict response to TG based on the discovery cohort. (A) Venn diagram representing common significantly changed transcripts from 28 coexpressed gene pairs and 57 differentially expressed genes, which were the RA therapeutic targets, as obtained from the DrugBank database. (B) The 17 major gene submodule constructed using the direct interactions among those genes. Red and blue circle nodes represent lncRNAs that were upregulated and downregulated, respectively. The node sizes represent the fold‐change in gene expression level in ascending order. The yellow lines represent co‐expression content. (C) The levels of Lnc‐ENST00000602558 and IGF1 expression were detected by quantitative PCR analysis. Each dot shows the expression levels of candidate genes in each individual patient (*n* = 25 and 15 for nonresponder and responder groups in the validation cohort, respectively). 18S and GAPDH were the internal reference. Error bars represent the standard error of the mean. ****p < .0001. GAPDH, glyceraldehyde phosphate dehydrogenase; IGF1, insulin‐like growth factor 1; lncRNAs, long noncoding RNAs.

A Pearson analysis of lncRNA/mRNA pairs was preformed to identify candidate genes that predicted a patient's response to TG treatment. The correlation analysis identified 422 and 451 pairs of lncRNA/mRNA pairs in the effective and poor efficacy groups, respectively. In addition, 28 pairs of lncRNA–mRNAs that showed a significant correlation (*p* < .05) were further screened in both the effective group and poor efficacy group. According to the database of DRUGBANK, the 57 differentially expressed genes were subsequently selected from the DrugBank database. The selected candidate genes were functionally involved into the signal pathways associated with major pathological events during RA progression, such as “inflammatory cell infiltration”, “inflammation,” “synovial pannus formation,” “angiogenesis,” “joint destruction,” and “bone resorption,” as well as “drug metabolism.” Considering their significantly differential expression patterns, great network topological importance, and functional relevance to RA, we selected these genes as the candidate gene biomarkers (Figure 2A). Subsequently, a gene signal transduction network consisting of 17 pair‐expressed genes was constructed. Following the calculation, Lnc‐ENST00000602558/IGF1 was identified as the most highly correlated coexpression gene pair in the gene‐gene interaction network associated with response to TG treatment (Figure 2B).

After identifying Lnc‐ENST00000602558/IGF1 as a candidate, reverse transcriptional quantitative PCR analysis was used to validate the microarray data by using the validation cohort of 40 patients, which consisted of 25 patients who responded and 15 patients who did not respond to TG treatment. Consistent with the result of microarray data, the levels of IGF1 expression in peripheral blood obtained from the responders were significantly lower than those in peripheral blood obtained from the non‐responders, as determined by using two internal references, S18 and GAPDH (Figure 2C). In addition, the levels of Lnc‐ENST00000602558 expression were markedly higher in the peripheral blood of responders than in the peripheral blood of non‐responders, which was also consistent with the microarray data (Figure 2D) (Table 1).

**Table 1:** Clinical and laboratory parameters of RA patients enrolled in the current study.

Parameters	Discovery cohort (*n* = 12)	Validation cohort (*n* = 40)
Age (years, mean SD)	47.8 ± 12.1	57.2 ± 7.8
Gender (male/female)	3/9	15/25
Disease duration (months, mean SD)	36.5 ± 12.8	45.6 ± 8.9
Erythrocyte sedimentation rate (ESR, mm/H, mean SD)	66.7 ± 29.6	36.4 ± 19.8
C‐reactive protein (CRP, mg/dL, mean SD)	25.6 ± 19.8	19.8 ± 20.1
Positive rheumatoid factor (*n*, %)	10, 77.5	24, 65.5
Positive anti‐cyclic citrullinated peptide (CCP) antibodies (*n*,%)	9, 65	24, 66

Abbreviations: RA, rheumatoid arthritis; SD, standard deviation.

### 3.3 The PLS‐based model efficiently predicted a patient's response to TG

**Figure 3:**
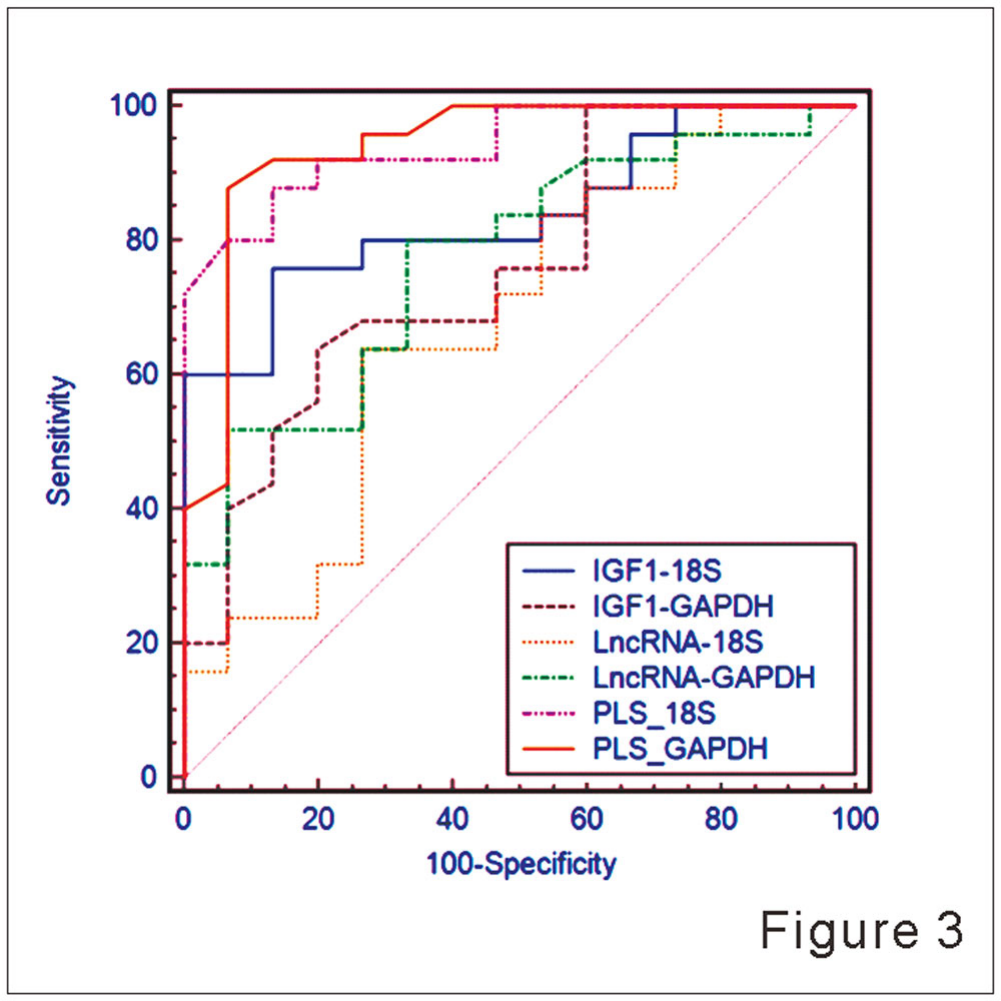
ROC comparisons of Lnc‐ENST00000602558, IGF1, and Lnc‐ENST00000602558/IGF1 using the PLS model. ROC curves were constructed for Lnc‐ENST00000602558, IGF1, and Lnc‐ENST00000602558/IGF1 using 18s and GAPDH as internal controls, as well as the PLS‐based model for predicting response to TG tablets. GAPDH, glyceraldehyde phosphate dehydrogenase; IGF1, insulin‐like growth factor 1; PLS, partial least squares; ROC, receiver operating characteristic.

To further evaluate the role of the ENST00000602558/IGF1 axis in predicting a patient's response to TG treatment, a partial least square (PLS)‐based model that used the expression levels of ENST00000602558 and IGF1 in peripheral blood was constructed. The performance of the model was validated by testing the levels of ENST00000602558 and IGF1 expression in samples of RA peripheral blood (normalized by 18s and GAPDH, respectively). The weight values of ENST00000602558 and IGF were first determined. When using 18s as the internal reference, the weight values of ENST00000602558 and IGF1 were −0.8251 and 0.5650, respectively, and the threshold value was −0.07. In addition, the weight values of ENST00000602558 and IGF1 were −0.7780 and 0.6282, respectively, and the threshold value was −0.035, when GAPDH served as the internal reference (Table 2). Subsequently, the high reliability and accuracy of the PLS model using the ENST00000602558/IGF1 axis was further confirmed by comparison with PLS models that used IGF1 or lncRNA expression alone. The result showed that the accuracy and AUC values of the PLS‐based model based on ENST00000602558 or IGF1 expression were 84% and 67.7%, respectively, when 18s served as the internal control. This was consistent with the PLS‐based model that used GAPDH as an internal control (the ENST00000602558 and IGF1 accuracy and AUC values were 94% and 75.9%, respectively). (Figure 3 and Table 3). As shown in Table 4, the PLS model based on ENST00000602558 or IGF1 expression alone was less accurate in predicting a patient's response to TG tablets when compared to the PLS‐based model, which was constructed based on the ENST00000602558/IGF1axis and used both GAPDH and RPS18 as internal controls. In summary, the reliability and efficacy of the PLS model that incorporated both IGF1 and lncRNA expression were significantly higher than those of the PLS model, which used either IGF1 or lncRNA alone.

**Table 2:** The weight values and threshold of Lnc‐ENST00000602558 and IGF1.

Groups	Weight values	Threshold
IGF1 (18s)	0.5650	−0.07
lncRNA (18s)	−0.8251	−0.07
IGF1 (GAPDH)	0.6282	−0.035
lncRNA (18s)	−0.7780	−0.035

Abbreviations: GAPDH, glyceraldehyde phosphate dehydrogenase; IGF1, insulin‐like growth factor 1; lncRNA, long noncoding RNA.

**Table 3:** AUC values result of Lnc‐ENST00000602558, IGF1, and Lnc‐ENST00000602558/IGF1 using 18s and GAPDH as an internal control, as well as the PLS‐based model in predicting response to TG tablets.

Groups	AUC values	SE values	95% Confidence interval
IGF1 (18s)	0.84	0.0705	0.69–0.936
lncRNA (18s)	0.677	0.0854	0.511–0.816
PLS (18s)	0.941	0.0818	0.818–0.99
IGF1 (GAPDH)	0.759	0.0829	0.594–0.879
lncRNA (18s)	0.772	0.0736	0.612–0.899
PLS (GAPDH)	0.940	0.0371	0.816–0.989

Abbreviations: AUC, area under the curve; GAPDH, glyceraldehyde phosphate dehydrogenase; IGF1, insulin‐like growth factor 1; lncRNA, long noncoding RNA; PLS, partial‐least‐squares; ROC, receiver operating characteristic; TG, tripterygium glycoside.

**Table 4:** ROC comparison of the Lnc‐ENST00000602558, IGF1, and Lnc‐ENST00000602558/IGF1 with the PLS‐based model.

Groups	Weight values	Threshold
IGF1 vs. PLS (18s)	0.84 vs. 0.94	0.056
lncRNA vs. PLS (18s)	0.667 vs. 0.94	0.002
IGF1 vs. PLS (GAPDH)	0.76 vs. 0.941	0.015
LncRNA vs. PLS (GAPDH)	0.772 vs. 0.941	0.018

Abbreviations: GAPDH, glyceraldehyde phosphate dehydrogenase; IGF1, insulin‐like growth factor 1; lncRNA, long noncoding RNA; PLS, partial‐least‐squares.

### 3.4 Treatment with TG increased Lnc‐ENST00000602558 expression and decreased IGF1 expression in TNF‐a‐induced MH7A cells

**Figure 4:**
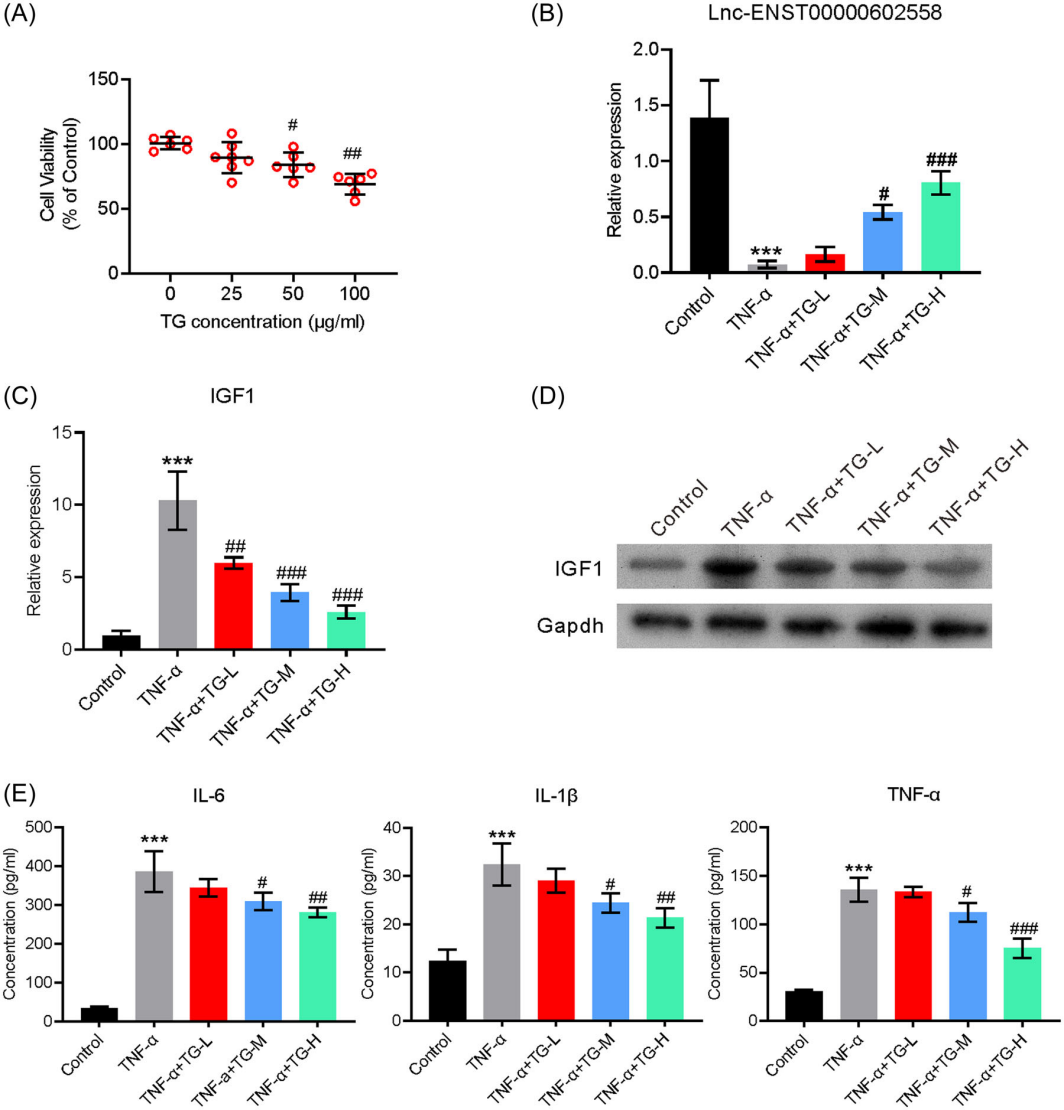
TG repressed TNF‐α induced IGF1 expression in MH7A cells. (A) Viability analysis of MH7A cells treated with different concentrations of TG. (B) MH7A cells were treated with TNF‐α and then incubated with 25 μg/mL (low dose, TG‐L), 50 μg/mL (medium dose, TG‐M) or 100 μg/mL TG (high dose, TG‐H). The levels of Lnc‐ENST00000602558 mRNA and IGF1 mRNA in MH7A cells after the indicated treatment. (C and D) The levels of IGF1 protein in MH7A cells after the indicated treatment. (E) The levels of IL‐6, IL‐1β, and TNF‐α in the above culture supernatants. Error bars represent standard error of the mean. *^(#)^ *p* < .05, **^(##)^ *p* < .01, ***^(###)^ *p* < .001. * represents control groups compared with control groups, # represents TNF‐α groups compared with TG groups. One‐way ANOVA was used in (A), (B), (C), and (E). The data were analyzed using Tukey's post hoc test with a confidence interval of 95% after ANOVA. ANOVA, analysis of variance; IGF1, insulin‐like growth factor 1; IL‐6, interleukin‐6; IL‐1β, interleukin‐1β; TG, tripterygium glycoside; TNF‐α, tumor necrosis factor‐α.

In an attempt to understand the effect of TG on IGF1 expression during RA development. MH7A cells were examined for their levels of IGF1 expression. MH7A cells were treated with different concentrations of TG for 24 h to evaluate the toxicity of the drug. Our data showed that a TG concentration < 100 µg/mL had little influence on cell survival (Figure 4A). To investigate the effect of TG on Lnc‐ENST00000602558 and IGF1 expression, the MH7A cells were treated with TNF‐α for 24 h to mimic a cell rheumatoid environment; after which, the cells were incubated with 25 μg mL (low dose, TG‐L), 50 μg/mL (medium dose, TG‐M) or 100 μg/mL of TG (high dose, TG‐H) for an additional 24 h. Subsequent RT‐qPCR analyses showed that treatment with TNF‐α alone significantly reduced Lnc‐ENST00000602558 expression. In contrast, the levels of IGF1, which plays an important role in RA development, were markedly increased in MH7A cells treated with TNF‐α. However, TG reversed the TNF‐α‐induced decrease in Lnc‐ENST00000602558 levels and increased the levels of IGF1 in a dose‐dependent manner (Figure 4B). Additionally, a western blot analysis showed that TG reduced the increase in IGF1 protein levels induced by TNF‐α (Figure 4C). An increased IGF1 level is reported to cause the secretion of pro‐inflammatory factors. In line with that premise, the levels of pro‐inflammatory factors in the supernatants of differently treated MH7A cells were examined by ELISA. The results showed that TG markedly reduced the levels of IL‐1β, IL‐6, and TNF‐α in the cell supernatants (Figure 4D,E). In summary, the results suggested that TG could regulate Lnc‐ENST00000602558/IGF1 expression and decrease TNF‐α‐induced inflammation in MH7A cells.

### 3.5 TG decreased IGF1 expression in an Lnc‐ENST00000602558‐dependent manner

**Figure 5:**
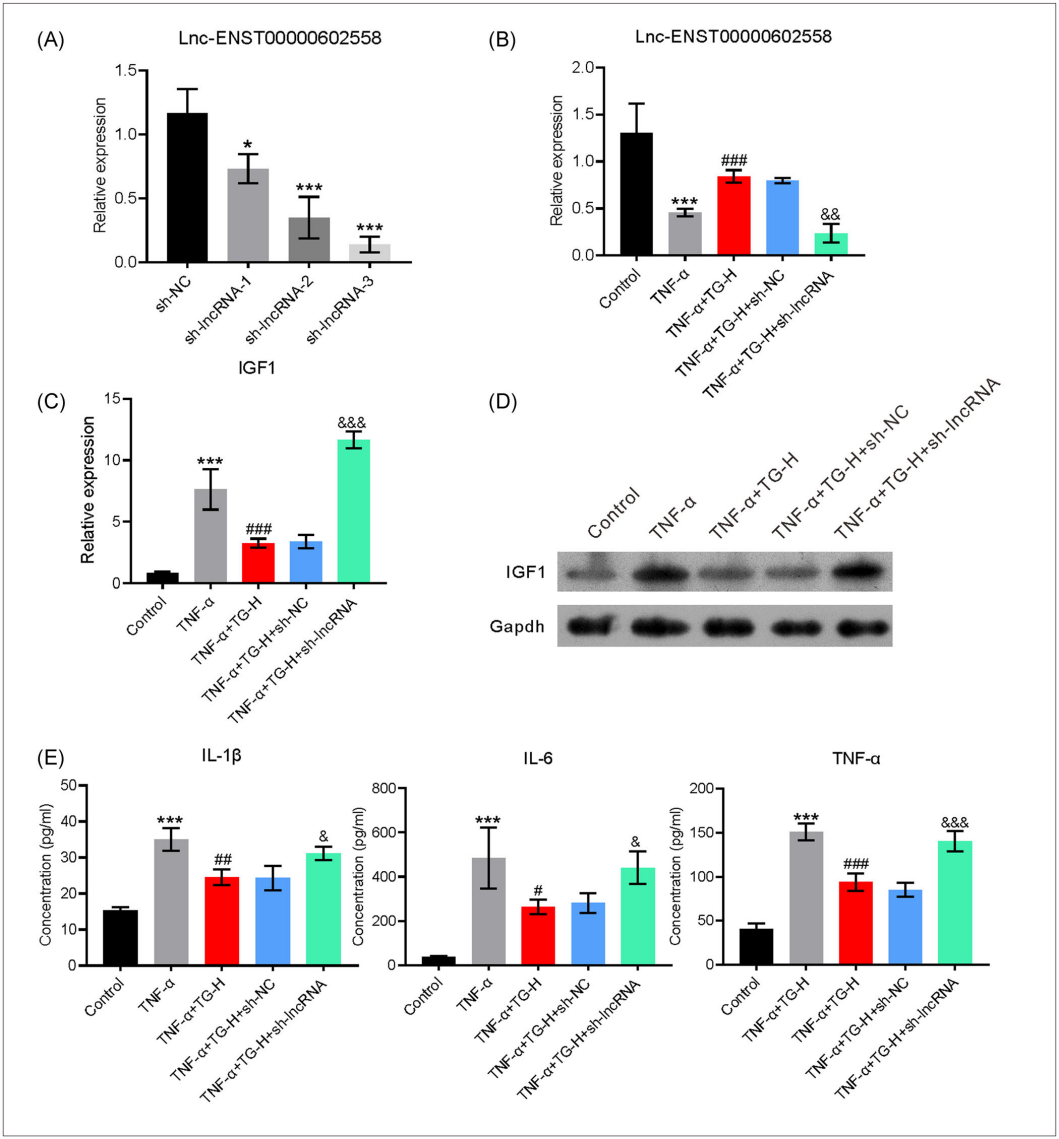
TG decreased IGF1 expression in an Lnc‐ENST00000602558‐dependent manner. (A) The levels of Lnc‐ENST00000602558 mRNA in MH7A cells treated with specific shRNAs. (B) MH7A cells were transfected with sh‐NC or sh‐LncRNA and then cultured for 24 h. The MH7A cells were then treated with different concentrations of TG, followed by incubation with TNF‐α. The levels of Lnc‐ENST00000602558 and IGF1 expression in MH7A cells after the indicated treatment. (C and D) The levels of IGF1 protein in MH7A cells after the indicated treatment. (E) The levels of IL‐6, IL‐1β, and TNF‐α in the above culture supernatants. Error bars represent standard error of the mean. *^(#,&)^ *p* < .05, **^(##,&&)^ *p* < .01, ***^(###,&&&)^ *p* < .001. *Represents control groups compared with control groups, #represents TNF‐α + TG groups compared with TNF‐α groups, &represents TNF‐α + TG + sh‐LncRNA groups compared with TNF‐α + TG + sh‐NC groups. One‐way ANOVA was used in (A), (B), (C), and (E). The data were analyzed using Tukey's post hoc test with a confidence interval of 95% after ANOVA. ANOVA, analysis of variance; IGF1, insulin‐like growth factor 1; IL‐6, interleukin‐6; IL‐1β, interleukin‐1β; TG, tripterygium glycoside; TNF‐α, tumor necrosis factor‐α.

To determine whether Lnc‐ENST00000602558 is necessary for TG‐mediated IGF1 expression, 3 pairs of shRNAs were designed and used to knockdown Lnc‐ENST00000602558 expression in MH7A cells. RT‐qPCR analyses showed that the sh‐LncRNA‐3 effectively decreased Lnc‐ENST00000602558 expression in the cells, and was used for subsequent studies (Figure 5A). MH7A cells were transfected with sh‐NC or sh‐LncRNA and then cultured for 24 h. Next, the MH7A cells were treated with different concentrations of TG, followed by incubation with TNF‐α. We found that treatment of the cells with TNF‐α decreased Lnc‐ENST00000602558 expression; however, that effect was attenuated when the cells were treated with TG. In addition, decreased expression of Lnc‐ENST00000602558 caused by sh‐LncRNA was also noted in MH7A cells treated with TNF‐α and TG (Figure 5B). Similarly, IGF1 expression was increased in MH7A cells treated with TNF‐α, and then became decreased after treatment with TG. As hypothesized, sh‐LncRNA significantly attenuated the TG‐induced decrease in IGF1 expression at both the mRNA and protein level in MH7A cells treated with TNF‐α (Figures 5C,D). To further examine the effect of Lnc‐ENST00000602558 expression on the function of TG, the levels of secretory pro‐inflammatory factor levels secreted from MH7A cells were analyzed by ELISA. Those results showed that the levels of IL‐1β, IL‐6, and TNF‐α expression in the supernatants of MH7A cells treated with TNF‐α were significantly higher the those in the supernatants of control cells. However, the levels of all those cytokines were significantly decreased following TG treatment. Consistent with the changes in IGF1 expression, ELISA results revealed that the concentrations of IL‐1β, IL‐6, and TNF‐α in the culture media of the sh‐RNA groups were significantly higher than those in the culture media of the sh‐NC groups treated with TNF‐α and TG (Figure 5E). Collectively, these results suggested that the effect of TG on the TNF‐a‐induced rheumatism cell model was impeded by Lnc‐ENST00000602558, as indicated increased IGF1 levels and the induced production of pro‐inflammatory cytokines.

### 3.6 Lnc‐ENST00000602558 directly regulated IGF1 expression

**Figure 6:**
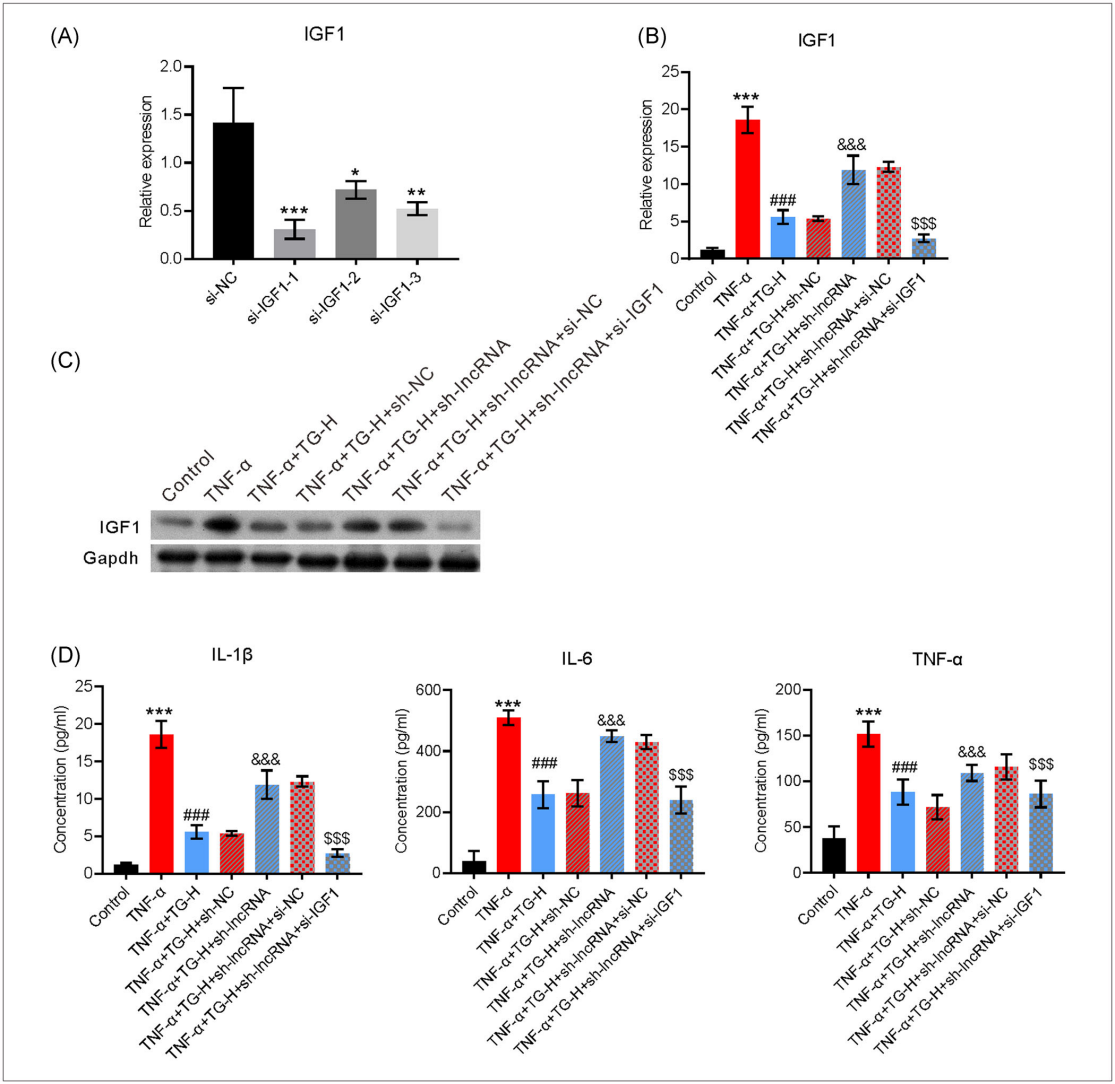
Lnc‐ENST00000602558 directly regulated IGF1 expression. (A) The levels of IGF1 mRNA in MH7A cells treated with specific siRNAs. (B) MH7A cells were transfected with sh‐LncRNA or si‐IGF1 and then cultured for 24 h. The MH7A cells were then treated with different concentrations of TG, followed by incubation with TNF‐α. The levels of IGF1 mRNA in MH7A cells after the indicated treatment. (C and D) The levels of IGF1 protein in MH7A cells after the indicated treatment. (E) The levels of IL‐6, IL‐1β, and TNF‐α in the above culture supernatant supernatants. Error bars represent standard error of the mean. *^(#, &, $)^ *p* < .05, **^(##, &&, $$)^ *p* < .01, ***^(###, &&&, $$$)^ *p* < .001. * represents control groups compared with control groups, # represents TNF‐α groups compared with TG groups. & represents TNF‐α + TG + sh‐LncRNA groups compared with TNF‐α + TG + sh‐NC groups. $ represents TNF‐α + TG + sh‐LncRNA + si‐IGF1 groups compared with TNF‐α + TG + sh‐LncRNA + si‐NC groups. One‐way ANOVA was used in (A), (B), (C), and (E). The data were analyzed using Tukey's post hoc test with a confidence interval of 95% after ANOVA. ANOVA, analysis of variance; IGF1, insulin‐like growth factor 1; IL‐6, interleukin‐6; IL‐1β, interleukin‐1β; TG, tripterygium glycoside; TNF‐α, tumor necrosis factor‐α.

To determine the underlying mechanism of Lnc‐ENST00000602558 in IGF1‐induced secretion of pro‐inflammatory factors in the RA cell model, IGF1 expression was systematically knocked down by using 3 pairs IGF1 specific siRNAs. RT‐qPCR results confirmed that IGF1 mRNA was expressed at low levels in the MH7A cells transfected with si‐IGF1‐1, and thus si‐IGF‐1 was used for subsequent studies (Figure 6A). Similarly, it was demonstrated that TG decreased IGF1 expression in MH7A cells treated with TNF‐α; however, knockdown of Lnc‐ENST00000602558 expression notably attenuated the effect of TG on IGF1 expression. Moreover, the levels of IGF1 mRNA and protein were significantly decreased in MH7A cells that had been treated with TNF‐α and TG and then transfected with si‐IGF1 and sh‐Lnc‐ENST00000602558 (Figures 6B,C). In addition, the levels of inflammatory cytokines were analyzed and the results revealed that knockdown of Lnc‐ENST00000602558 expression in MH7A cells significantly restored the levels of IL‐1β, IL‐6, and TNF‐α following treatment with TNF‐α and TG. However, transfection with si‐IGF1 significantly decreased IGF1 expression in MH7A cells that had been treated with TNF‐α and TG and then transfected with sh‐LncRNA (Figure 6D). Therefore, these results suggested that Lnc‐ENST00000602558 suppressed the secretion of inflammatory cytokines from MH7A cells by regulating IGF1 expression after the cells had been treated with TNF‐α and TG.

**Figure 7:**
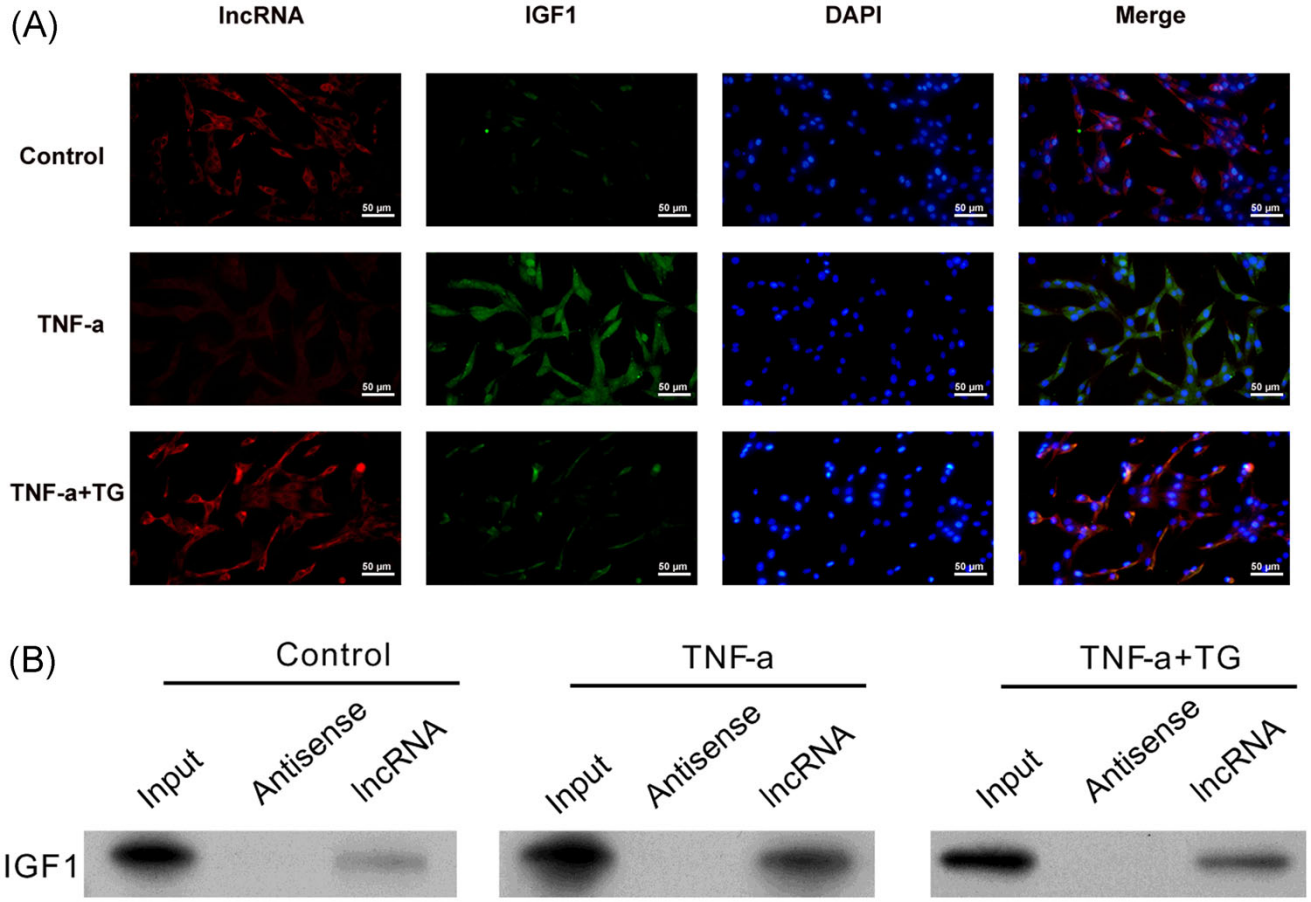
Lnc‐ENST00000602558 directly regulated IGF1 expression. (A) FISH analysis of the interaction between Lnc‐ENST00000602558 and IGF1. Red fluorescence (TRITC) represents Lnc‐ENST00000602558, green fluorescence (FITC) represents IGF1, and blue fluorescence (DAPI) represents the cell nucleus. (B) RNA pull down analysis of the interaction between Lnc‐ENST00000602558 and IGF1 in the MH7A cell extracts. DAPI, 4′,6‐diamidino‐2‐phenylindole; FISH, fluorescent in situ hybridization; IGF1, insulin‐like growth factor 1; TG, tripterygium glycoside; TNF‐α, tumor necrosis factor‐α.

A previous study reported that lnc‐RNAs regulates gene expression by directly interacting with their target proteins (23). Our data proved that Lnc‐ENST00000602558 interrupted TNF‐α‐induced IGF1 expression at the transcriptional level. To further confirm whether Lnc‐ENST00000602558 induced IGF1 expression via direct interaction with IGF1, a fluorescence in situ hybridization (FISH) analysis was performed. The FISH results showed that the fluorescence intensity of Lnc‐ENST00000602558 almost disappeared after TNF‐α treatment. In contrast, treatment with TG restored the fluorescence intensity of Lnc‐ENST00000602558 when compared to cells treated with TNF‐α. In addition, when compared with the TNF‐α treatment group, the fluorescence intensity of IGF1 was decreased in MH7A cells treated with TNF‐α and TG, and redistribution of IGF1 expression was especially profuse in the Lnc‐ENST00000602558 regions, suggesting that Lnc‐ENST00000602558 might directly interact with IGF1 and thereby regulate that protein's stability (Figure 7A). RNA pull down assays also confirmed that Lnc‐ENST00000602558 was able to bind with the IGF1 protein in TNF‐α‐induced MH7A cells, and TG treatment diminished the amount of IGF1 protein that bound to Lnc‐ENST00000602558 (Figure 7B). These findings, when combined with the abnormal secretion of pro‐inflammatory factors observed in TG‐ and sh‐RNA‐treated MH7A cells, indicated that Lnc‐ENST00000602558 participates in the therapeutic effect of TG, at least partially, by directly interacting with IGF1 protein.

## 4 DISCUSSION

TG has been widely used to treat RA,^28^, ^29^ and has attracted extensive attention due to its strong efficacy.^15^, ^30^, ^31^ However, not all RA patients benefit to the same degree from TG treatment.^14^, ^32^

*T. wilfordii* (TwHF) preparations affect various human systems, including the digestive, reproductive, and cardiovascular systems. The most common manifestations of side effects by TwHF preparation is the digestive system. Oral taken of TwHF caused local irritation of the gastrointestinal mucosa and some mild symptoms, such as dry mouth, fatigue, and loss of appetite.^33^ TwHF can also induce rash, skin pigmentation, skin itching, oral ulcers, and hair loss in skin and its accessories.^34^, ^35^ Besides, chronic treatment of TwHF also influence the reproductive systems,^36^, ^37^, ^38^ hepatotoxicity,^39^, ^40^ the blood and hematopoietic system,^41^, ^42^ and cardiovascular system.^43^ TwHF also causes dizziness, lethargy, insomnia, neuritis, diplopia, and other central and peripheral nervous system toxicity. Some other ADRs, including rash, itching, hair loss, and facial pigmentation, have occasionally been observed in different individuals.^44^ Hence, the side effects should be monitored regularly during the clinical use of TwHF preparations.

TG tablets, as the main effective ingredients of *T. wilfordii*, are the most commonly used TwHF‐based therapy and display better therapeutic effects than several modifying antirheumatic drugs according to recent clinical observations.^20^ However, due to the limited number of long‐term clinical trials, it is difficult to make a comprehensive evaluation of the efficacy of TG preparations in treating RA. Besides, the pathogenesis and etiology of RA and the exact mechanism of action of TG preparations have not been fully clarified, which seriously hindering the wide acceptance of this preparation in countries other than China and the individualized treatment of RA patients. Moreover, TG and its extracts contain a variety of chemical components that may have synergistic and/or antagonistic effects.^45^ Overall, approximately 30% of RA patients treated with TG tablets fail to achieve clinical improvement.^46^, ^47^

To explain this issue, the whole genome expression profile of RA patients treated with TG tablets was investigated to identify gene pairs that were differentially expressed and might to predict a patient's response to TG treatment. Differentially expressed lncRNAs–mRNAs pairs were obtained and a co‐expression regulation network was used to search for the individual therapeutic effect of TG treatment. Based on the candidates obtained, the Lnc‐ENST00000602558/IGF1 axis, which showed significant differential expression among a series of different lncRNAs and mRNAs, was selected for our study. We then constructed models that were used to predict therapeutic efficacy in RA based on the expression of Lnc‐ENST00000602558, IGF1, and Lnc‐ENST00000602558/GF1 pairs. Our results showed that when compared with a model based on either IGF1 or Lnc‐ENST00000602558 expression alone, a model based on both Lnc‐ENST00000602558 and IGF1 expression had a high predictive accuracy and area under ROC curve. More importantly, our new PLS model based on the expression of Lnc‐ENST00000602558/IGF1 pairs revealed the need for a deeper understanding of how molecular regulatory networks function in response to therapy.

This study examined individual differences in RA patients that might affect their response to treatment with TG tablets. Our findings showed that RA patients have certain individual biochemical characteristics that greatly affect their response to TG. Several differentially expressed lncRNA–mRNA pairs were identified, and a regulated network of lncRNA–mRNA co‐expression was used to identify individual differences that affected the efficacy of TG when used to treat RA patients. Thus, the process used to select drugs can be improved by taking individual patient characteristics into account, as it will help patients achieve the best therapeutic effect, while minimizing drug toxicity and cost. Our current study was conducted using RA patients rather than an animal disease model or in vitro cell culture model. We examined how lncRNA–mRNA expression was related to individual differences in the efficacy of TG in treatment of RA. We also established a molecular prediction model based on lncRNA that could be used for the personalized treatment of RA with TG. Our results bring us closer to the use personalized RA treatment in the clinic.

Subsequently, cellular functional tests were performed to further verify the regulatory effect of TG on the Lnc‐ENST00000602558/IGF1 axis, and to confirm whether the clinical efficacy of TG is related to the gene pair. During in vitro studies, increased IGF1 expression, which is the primary mediator of inflammation,^18^, ^48^ was noted upon TNF‐α challenge. Here, we revealed that Lnc‐ENST00000602558 could reduce TNF‐α‐induced IGF1 expression. Furthermore, IGF1 expression was increased in MH7A cells when Lnc‐ENST00000602558 was expression suppressed by transfection with sh‐LncRNA‐3, indicating that Lnc‐ENST00000602558 was a potent inhibitor of inflammation and acted by regulating IGF1. To help verify that hypothesis, we respectively knocked down Lnc‐ENST00000602558 and IGF1 expression in MH7A cells, and subsequently evaluated the secretion of pro‐inflammatory factors after treatment with TNF‐α and TG. As expected, the inhibitory effect of TG on secretion of pro‐inflammatory factors by MH7A cells was significantly blocked when Lnc‐ENST00000602558 expression was knocked down. Nevertheless, Lnc‐ENST00000602558 had little influence on inflammation when IGF1 was knocked down in MH7A cells, suggesting that Lnc‐ENST00000602558 primarily regulates RA via IGF1 expression and a downstream pathway.

Our study also revealed the role of Lnc‐ENST00000602558 as an important regulator of the IGF1 signaling pathway. To date, the transcriptional regulation of IGF1 expression and pro‐inflammatory factor secretion has largely been attributed to the IGF1 transcriptional and translation module, which plays an integrative role in relaying physiological signals to a key subset of transcription factors that direct and coordinate the expression of pro‐inflammatory genes.^22^, ^49^ Our data demonstrate that Lnc‐ENST00000602558 is an essential regulator of this transcriptional module, and the basis for this conclusion is manifold. First, Lnc‐ENST00000602558 deficiency in MH7A cells shows striking parallels to the phenotype observed in IGF1 overexpressing MH7A cells, including altered expression of pro‐inflammatory factor genes and changes in the secretion of pro‐inflammatory factors. In addition, IGF1 knockdown restored the impaired inflammatory effects caused by Lnc‐ENST00000602558 depletion, including altered gene expression and secretion of pro‐inflammatory factors. Finally, an in vitro FISH analysis revealed that Lnc‐ENST00000602558 directly interacts with the IGF1 protein. The mechanistic studies presented here suggest that inhibition of a pro‐inflammatory effect can occur independent of canonical signaling events, as lncRNA directly binds with its regulator in macrophages. It is conceivable that the mechanisms utilized by Lnc‐ENST00000602558 might be more complex than the model we propose.

As a lymphohemopoietic cytokine, IGF‐1 has profound positive effects on immune function.^50^ IGF‐1 binds to the T and B cells and induces anti‐CD3‐stimulated T cell proliferation.^51^ It also activates T cell Akt, thereby enhancing lymphocyte survival.^52^ Recently, IGF‐1 reportedly prevents cord blood T cells from spontaneous apoptosis when cultured in a serum‐free medium.^53^ Hence, IGF‐1 influences the onset of inflammatory diseases, thereby, the abundance and profile of IGF‐1 are used to serve as important determinants of signaling of RA. A recent study showed that the IGF‐1 levels in the serum and synovial fluid were significantly lower in patients with RA.^54^ However, IGF‐1 have limitations in reflecting clinical symptoms such as joint swelling or tenderness when evaluating disease activity of patients with RA. Therefore, in the present study, we investigated the Lnc‐ENST00000602558/IGF1 axis to assess the biomarkers between responsive and nonresponsive RA patients to TG tablets and to identify the candidate gene biomarkers according to both the differential expression patterns and the network topological features.

The study reveals a previously undiscovered function of a signaling network consisting of Lnc‐ENST00000602558 and IGF1 in RA patients treated with TG. Here, we systematically integrated microarray data generated by a differential gene expression analysis with the topological features of a gene signal transduction network. Our mechanistic studies revealed a pathway which regulates IGF1 expression via Lnc‐ENST00000602558. Additionally, our results indicate a possible role for the Lnc‐ENST00000602558/IGF1 axis in regulating the expression and secretion of pro‐inflammatory factors. Our proposed molecular mechanism suggests that MH7A cell‐derived Lnc‐ENST00000602558 acts as a novel and important factor that improves the therapeutic effect of TG on RA by inhibiting IGF1 expression. These findings increase our understanding of various molecular aspects of inflammation, and contribute to the development of strategies for predicting the therapeutic effect of TG treatment. In summary, we propose that in RA patients, increased levels Lnc‐ENST00000602558 caused by TG treatment may contribute to differences in clinical response to TG treatment. Beyond the established adverse effects of protracted corticosteroid use, the Lnc‐ENST00000602558/IGF1 axis may be a biomarker and molecular target for use in treatment of RA.

## AUTHOR CONTRIBUTIONS

Hailong Wang participated in design of the study. Yang Gao performed the data collection, analysis, and interpretation. Yanfeng Gao, Jian Bai, Yanpeng Zhao, Renyi Wang, Hanzhou Wang, Guangzhao Zhu, Xixi Wang, and Xiaochen Han helped in data collection. Xiaoyue Wang helped in data analysis. Yanqiong Zhang provided help in literature analysis. Yang Gao wrote the manuscript. All authors read and approved the manuscript.

## CONFLICTS OF INTEREST STATEMENT

The authors declare no conflicts of interest.

## ETHICS STATEMENT

The current study was performed according to the guidelines of the Declaration of Helsinki for humans. The clinical research was approved by the Research Ethics Committee of Guang'anmen Hospital (Beijing, China), and consent was obtained from all patients.

## Data Availability

The data that support the findings of this study are available from the corresponding authors upon reasonable request.
